# Comparative transcriptomic analysis reveals the molecular mechanism underlying seedling heterosis and its relationship with hybrid contemporary seeds DNA methylation in soybean

**DOI:** 10.3389/fpls.2024.1364284

**Published:** 2024-02-19

**Authors:** Xiaobo Ren, Liangyu Chen, Lin Deng, Qiuzhu Zhao, Dan Yao, Xueying Li, Weixuan Cong, Zhenyuan Zang, Dingyi Zhao, Miao Zhang, Songnan Yang, Jun Zhang

**Affiliations:** ^1^ Faculty of Agronomy, Jilin Agricultural University, Changchun, China; ^2^ Zhanjiang City Key Laboratory for Tropical Crops Genetic Improvement, South Subtropical Crops Institute, Chinese Academy of Tropical Agricultural Sciences, Zhanjiang, China; ^3^ College of Life Science, Jilin Agricultural University, Changchun, China; ^4^ National Crop Variety Approval and Characteristic Identification Station, Jilin Agricultural University, Changchun, China

**Keywords:** soybean, seedling heterosis, RNA-Seq, DNA methylation, contemporary hybrid seed

## Abstract

Heterosis is widely used in crop production, but phenotypic dominance and its underlying causes in soybeans, a significant grain and oil crop, remain a crucial yet unexplored issue. Here, the phenotypes and transcriptome profiles of three inbred lines and their resulting F_1_ seedlings were analyzed. The results suggest that F_1_ seedlings with superior heterosis in leaf size and biomass exhibited a more extensive recompilation in their transcriptional network and activated a greater number of genes compared to the parental lines. Furthermore, the transcriptional reprogramming observed in the four hybrid combinations was primarily non-additive, with dominant effects being more prevalent. Enrichment analysis of sets of differentially expressed genes, coupled with a weighted gene co-expression network analysis, has shown that the emergence of heterosis in seedlings can be attributed to genes related to circadian rhythms, photosynthesis, and starch synthesis. In addition, we combined DNA methylation data from previous immature seeds and observed similar recompilation patterns between DNA methylation and gene expression. We also found significant correlations between methylation levels of gene region and gene expression levels, as well as the discovery of 12 hub genes that shared or conflicted with their remodeling patterns. This suggests that DNA methylation in contemporary hybrid seeds have an impact on both the F_1_ seedling phenotype and gene expression to some extent. In conclusion, our study provides valuable insights into the molecular mechanisms of heterosis in soybean seedlings and its practical implications for selecting superior soybean varieties.

## 1 Introduction

Heterosis refers to a phenomenon where the F_1_ offsprings resulting from crosses between genetically diverse parents exhibit superior traits compared to both parents (Birchler et al., 2010; Chen, 2013). Despite its successful utilization in crop production, the underlying genetic basis of hybrid dominance remains unclear. Several dominant hypotheses have been proposed to explain its mechanisms, including the dominance hypothesis (Jones, 1917), the overdominance hypothesis (Shull, 1908), and the epistasis hypothesis (Rhode and Cruzan, 2005). These hypotheses focus on DNA differences between parents and attempt to determine the contribution of allelic and non-allelic loci to phenotypic dominance, but they do not provide a comprehensive explanation of the biological processes involved.

Changes in gene expression levels are better linked to a particular phenotype than genetic variation. Based on high-throughput sequencing technology, there has been an increasing number of studies in plants using transcriptome to resolve phenotypic heterosis. Most studies, including those on maize (Kwan et al., 2016; Ma et al., 2018), oilseed rape (Zhu et al., 2020), pigeonpea (Sinha et al., 2020), and *Arabidopsis* (Zhu et al., 2016; Liu et al., 2021), have shown that F_1_ hybrid seedlings with biomass dominance mainly exhibit expression levels higher than the parental mean, equal to the optimal parent, or higher than both parents in genes such as photosynthesis and photosynthetic product utilization. In contrast, F_1_ seedlings without traits advantage did not display such characteristics (Fujimoto et al., 2012; Xiong et al., 2022). Cytological observations have also suggested that leaf size in superior hybrid seedlings results from an increase in cell number or size, which is associated with an up-regulation of the expression of genes involved in relevant biological processes such as cell division (Li et al., 2021a; Hu et al., 2022). Furthermore, it has been discovered that circadian rhythm factors regulate alterations in the expression of target genes and are responsible for starch accumulation in F_1_ hybrid seedlings (Ni et al., 2009; Kwan et al., 2016; Wang et al., 2017). In terms of gene reprogramming pattern in F_1_, the study of temporal transcriptome mapping of *Arabidopsis* seedlings further revealed that hybrids exhibit parental selective inheritance in gene expression levels, i.e. key genes originating from different parents and controlling important biological processes are selected for expression in hybrids according to the time period of growth and development to form a strong complementary network (Liu et al., 2021). The above studies have demonstrated that the traits at specific growth stages combined with transcriptomic data can, to some extent, link phenotypic heterosis to specific biological processes. An ongoing debate is whether the main pattern of F_1_ hybrid gene expression is dominated by additive or non-additive, i.e. whether the gene expression abundance is mostly equal to the average of the parental expression levels. Most studies have illustrated that F_1_ hybrids are predominantly non-additive in expression and have a clear tendency to favor parental expression levels (Tong et al., 2013; Liu et al., 2021; Xiong et al., 2022). However, several studies in maize have supported the notion that additive expression is the dominant pattern of hybrid (Stupar et al., 2008; Ma et al., 2018; Zhou et al., 2019). Although the possible reasons for these problems are due to the application of different statistical methods, they also indicate that the expression profiles of hybrids are influenced by different species and growth periods. Therefore, unravelling the biological basis of phenotypic advantages requires a continuous complementation of transcriptome profiles across species, organs, and successive things to find the paradigm of heterosis.

DNA methylation does not alter the nucleotide base sequence and generally occurs on cytosines but can affect gene expression (Williams and Gehring, 2020; Nuñez et al., 2021). There are a total of three cytosine contexts in plants: CG, CHG (where H = A, T or C) and CHH, with the CG sites being the major type of methylation (Zhang et al., 2018). In *Arabidopsis* and pigeonpea, the pattern of DNA methylation changes is similar to that of gene expression in F_1_ seedlings, both of which are dominated by non-additive types and involve very similar biological pathways (Shen et al., 2012; Sinha et al., 2020). On the other hand, DNA methylation recombination profiles of contemporary seeds in rice hybrids are associated with non-additive expression of several rhythmic and yield-related genes (Zhou et al., 2021), and the methylation sites formed during the zygote period can be transferred to subsequent life processes (Liu et al., 2023). A fact that the rhythmic expression of the *AtCCA1* (*Circadian clock associated 1*) gene formed 10 days after cross-pollination can be maintained until late plant development and demethylation of the gene in hybrid seedlings results in the loss of biomass heterosis (Ng et al., 2014). Researchers hypothesized that DNA methylation or gene expression profiles during the same period as the phenotype are the result of hybrid advantage, and that methylation profiles formed during the contemporary seed of hybrid could be the result of heterosis (Zhou et al., 2021). Therefore, studying the relationship between epigenetic profiles of immature hybrids and gene expression during the subsequent growth period will help to analyze the initiation mechanism of hybrid dominance and may provide unique insights.

Soybean is a significant oilseed crop and faces challenges in meeting the demand for production, particularly in China and many developing countries (Karikari et al., 2020). Yield heterosis has been observed in soybeans, and these studies utilizing transcriptomic data have linked yield advantage to physiological rhythms, carbon fixation, amino acid metabolism, and other processes (Taliercio et al., 2017; Zhang et al., 2017). Using the methylation sensitive amplification polymorphism technique and MethylRAD-Seq, several studies have identified a link between the extent of the yield benefit and the degree of DNA methylation present in F_1_ leaves or contemporary hybrid seeds (Wang et al., 2018; Song et al., 2020). However, previous studies neither thoroughly examined the restructuring patterns of hybrid methylation or gene expression profiles, nor have they fully elucidated the underlying biological mechanisms of heterosis. It remains unclear whether the seedling phase, which is a crucial period in soybean growth, exhibits traits advantage and the molecular mechanisms behind this phenomenon stay unknown. Here, we specifically examined soybean seedlings from four F_1_ hybrid derived from two crossing combinations with varying yield heterosis that had been previously studied (Wang et al., 2018; Song et al., 2020). The focus will be on the recompilation of gene expression patterns within hybrids and the biological processes linked to phenotypic dominance. Additionally, DNA methylation data obtained from immature seeds in previous studies were re-analyzed to explore their correlation with seedling expression profiles. The ultimate goal of this research is to enhance our understanding of the molecular mechanisms driving plant heterosis, which, in turn, will facilitate the selection and utilization of soybean hybrid varieties.

## 2 Materials and methods

### 2.1 Soybean plant materials and growing environment

Based on previous studies (Song et al., 2020; Chen et al., 2022b), we used Jilin 38 (P1) as the shared parent soybean variety and crossed it artificially with Yi 3 (P2) and Jilin 47 (P3) at the experimental field of Jilin Agricultural University (Changchun, China) in 2021. This resulted in the formation of four hybrid combinations: F12 (P1 × P2, where the first parent is maternal and the second one is paternal), F21 (P2 × P1), F13 (P1 × P3), and F31 (P3 × P1) seeds. To investigate the performance of the two soybean hybrid combinations in seedling biomass and minimize the influence of external factors on seedling emergence and growth, the experiment was carried out following these steps: Initially, the seeds were cleaned by wiping their surface with cotton soaked in 75% alcohol, followed by rinsing with distilled water for 2 minutes. Next, plant pots were labeled with plant numbers on their sides. 200g of prepared nutrient soil [consisting of the soil (substrate soil, Pindstrup Mosebrug A/S, Denmark) and vermiculite in a 3:1 ratio] were poured into each pot. Each seed was sown in separate pot in 3 cm in depth of soil. Randomly, the pots containing the seeds were placed in plastic trays in the artificial climatic chamber (RPX-1200A, YiXi, Shanghai, China). To prevent growth differences resulting from the placement of plants in different parts of the incubator, each column and row in the tray was planted with the same number of seven genotypes seeds. The incubation environment was set as follows: 16 hours of light and 8 hours of darkness, with a temperature of 25°C during the light period and 20°C during the dark period. The relative humidity was maintained at 70%, and the light intensity was set at 300 μmol/(m2-s). Distilled water was poured into the trays every 24 hours to ensure uniform water absorption by each pot and to keep the soil moist.

### 2.2 Determination of seedling phenotype for soybean

Six phenotypes were measured in the seedlings at 15 days after sowing (DAS 15), with a minimum of 15 seedlings examined for each genotype. Furthermore, the weight of 15 seeds from contemporary hybrid seeds was measured, and their fat and protein contents were determined using near-infrared spectroscopy (NIRFlex N-500, Buchi, Flawil, Switzerland). Each genotype had three biological replicates. Mid-Parent heterosis (MPH) and better-parent heterosis (BPH) were calculated as follows: MPH = [(mean value of F_1_ hybrid - average value of both parents)/average value of both parents] × 100%, BPH = [(mean value of F_1_ hybrid - mean value of optimal parent)/mean value of optimal parent]× 100%. Descriptive statistics and one-way analysis of variance (ANOVA) for these phenotypes were conducted using DPS v9.5 software (Tang and Zhang, 2013). Visual representation of the statistical results was achieved using R (r-project.org) version 4.1.2 and the R packages Hmisc, corrplot, ggplot2, pheatmap, and patchwork.

### 2.3 DNA isolation and F_1_ hybrid identification

Before conducting transcriptome sequencing, we ensured the authenticity of F_1_ seedlings. Randomly selecting the root of three seedlings of each genotype, including the parents, and DNA was extracted using the Plant Genomic DNA Kit (CW0553M, Cowin Biotech Co. Ltd., Beijing, China). After verifying the integrity of genomic DNA, InDel markers developed by genotyping-by-sequencing method (unpublished) were used to distinguish between parents and their F_1_ offspring (**Supplementary Table S1**). The experiment followed the instructions of the 2 × M5 HiPer plus Taq HiFi PCR mix (MF002-plus, Mei5 Biotechnology Co., Ltd., Beijing, China). The PCR reaction operated for 30 cycles with a system of 25 s at 94°C, 25 s at 59°C, and 30 s at 72°C. Finally, PCR products underwent electrophoresis using a 1.5% agarose gel and were identified by a UV gel imager (JY04S-3E, JunYi electrophoresis Co., Ltd., Beijing, China).

### 2.4 RNA isolation and RNA-Seq

After recording the phenotypic data, seedlings at DAS15 were promptly preserved in liquid nitrogen. Subsequently, a sequencing pool was created by combining 3-5 seedlings, and three biological replicates were generated based on their respective genotypes. Total mRNA from 21 samples were isolated and purified using TRIzol (15596018, Thermo Fisher, Waltham, MA, USA) following the manufacturer’s procedure. The quantity and purity of mRNA were assessed using a NanoDrop ND-1000 (Thermo Fisher, Waltham, MA, USA) and a Bioanalyzer 2100 (Agilent, Santa Clara, CA, USA). Library construction and RNA-seq were performed by the LC Biotechnology Company (Hangzhou, China), following the manufacturer’s protocol. The NovaseqTM 6000 (Illumina, San Diego, CA, USA) platform was utilized for sequencing, generating paired-end reads of 150 bp from all the libraries. Chean data from all samples were aligned by HISAT2 (Kim et al., 2019) to the reference genome Wm82.a2.v1 from Phytozome (Schmutz et al., 2010; Goodstein et al., 2012), the resulting data were used to calculate mRNA expression levels using StringTie (Pertea et al., 2015), and the expression levels were normalized to fragments-per-kilobase of transcript-per-million mapped reads (FPKM). Finally, a gene was discarded if the average FPKM <1 in all seven genotypes. All genes after filtering were used for subsequent bioinformatics analysis.

### 2.5 Bioinformatic analysis

Gene counts obtained from sequencing were directly inputted into DESeq2 for the analysis of differentially expressed genes (Love et al., 2014a, b). The differentially expressed genes (DEGs) were identified using a *p*-value < 0.05 and |log2(fold change)| > 1. All gene annotation has been obtained from Phytozome (Wm82.a2.v1). Gene ontology (GO) enrichment analysis was conducted using Clusterprofile 4.0 (Wu et al., 2021). The criteria for significantly enriched GO term were a *p*.adjust < 0.05 and gene count ≥ 5.

The weighted gene co-expression network analysis (WGCNA) was conducted following the methodology outlined by Liu et al. (Liu et al., 2021). Briefly, gene expression levels were transformed using log2 (FPKM+0.001) and subsequently filtered based on highest median absolute deviation. A soft threshold of 16 was selected by fitting the optimal scale-free topology model. Finally, a “signed” network was constructed in which gene modules were used to calculate relationships with seedling traits. Phenotypically significant modules were required to satisfy both that the correlation between eigengene and phenotype was *p <*0.05, and that the correlation between gene significance and module membership within the module was *p <*0.05. Analyses were done using the R package WGCNA (Langfelder and Horvath, 2008).

Methylation data from immature seeds was gathered from a preceding study (Song et al., 2020; Chen et al., 2022b), whereby the relative methylation levels [reads per million (RPM)] of all methylation sites were applied to compute the overall methylation amount within a specific genome area. Additionally, we employed Pearson correlation analysis to determine the correlation between gene methylation and expression. The type of methylation remodeling pattern of loci was classified based on the results of previous differential methylation analysis (Chen et al., 2022b), using the classification method of gene expression.

### 2.6 Quantitative reverse transcription PCR

The quality of the RNA-seq was assessed using all samples with qRT-PCR. First, the RNA samples were reverse transcribed to cDNA using Maxima Reverse Transcriptase (EP0743, Thermo Scientific, MA, USA). Next, the PCR mix was prepared using 2×RealStar Fast SYBR qPCR Mix (A304, GenStar, Beijing, China) reagents following the manufacturer’s instructions. Finally, Gene-specific primers (**Supplementary Table S2**) were applied to qRT-PCR using a LightCycler480 II PCR machine (Roche, Rotkreuz, Switzerland). The actin gene (*Glyma.18G290800*) was used as the housekeeping gene, and the 2^-ΔΔCt^ method with three biological replications was performed for analysis (Livak and Schmittgen, 2001).

## 3 Results

### 3.1 Phenotypic analysis of hybrids and parents

**Figure 1:**
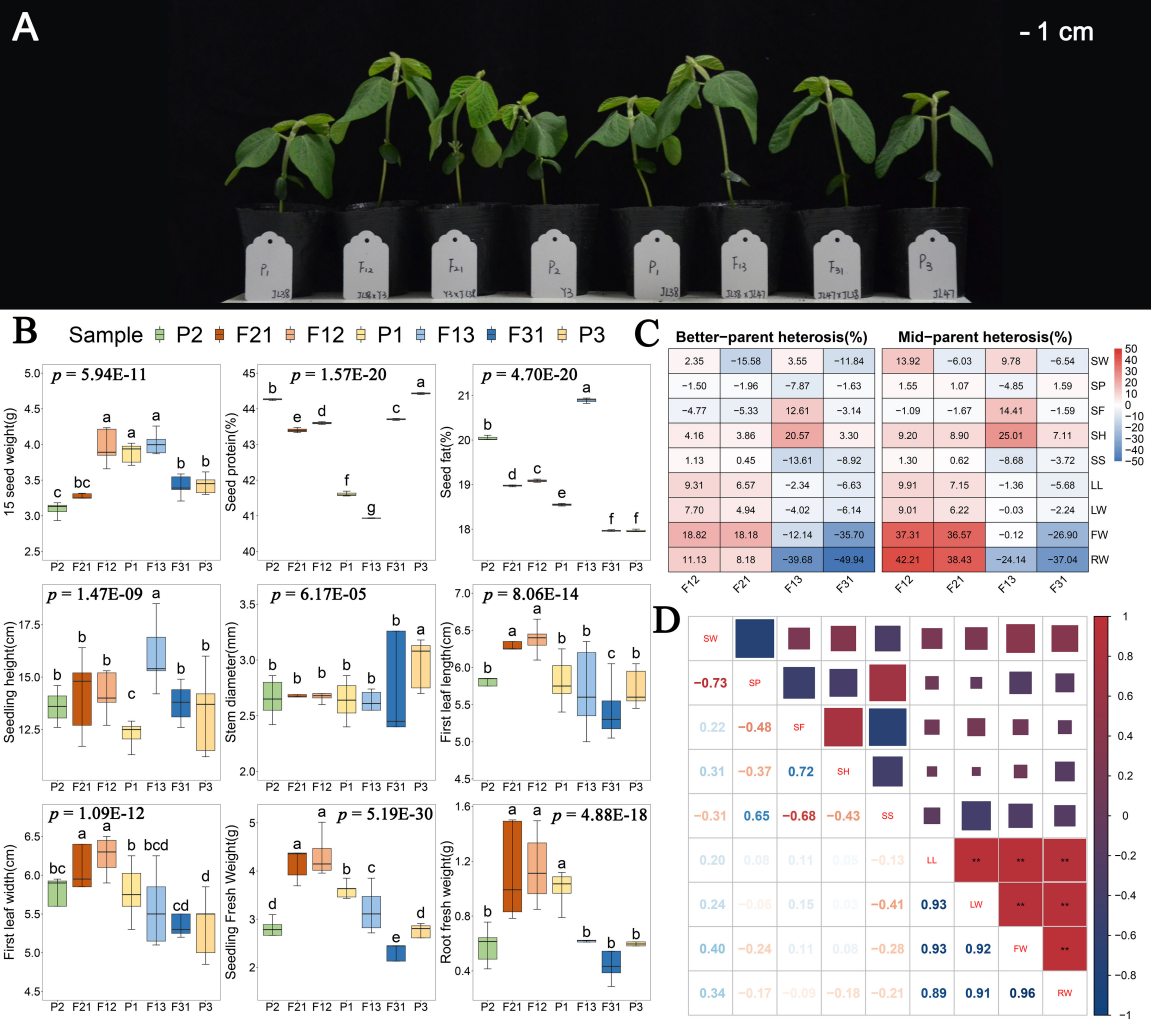
Phenotypic analyses of F_1_ and their parental seedlings. **(A)** Soybean seedling growth of all hybrids and their parents; **(B)** One-way ANOVA analysis of seedling traits. The *p*-values displayed on the figure represent the results of the phenotypic ANOVA. The lowercase characters on the bar indicate *p* < 0.05 for the results of multiple comparisons after ANOVA; **(C)** Heterosis index of four hybrids combinations in all traits; **(D)** Pearson’s Correlation analysis of seedling phenotype. ^**^ indicate *p* < 0.01.

In this study, four F_1_ hybrid lines were generated through reciprocal artificial crossing of Yi 3(P2) and Jilin 47 (P3), with Jilin 38 (P1) served as a common parent. The authenticity of these hybrids was confirmed using InDel markers (**Supplementary Figure S1**). Morphological observations (**Figure 1A**) and the results of one-way analysis of variance for phenotypes demonstrated significant genotype influence (*p* < 0.01) on seedling height, stem thickness, first leaf length, first leaf width, seedling fresh weight, and root fresh weight at DAS15 (**Figure 1B**). Furthermore, the heterosis index indicated that the hybrids resulting from the P1 × P2 combination exhibited positive phenotypic dominance in all six traits (**Figure 1C**). Particularly, seedling and root fresh weights demonstrated an increase of more than 8% compared to the optimal parents, with the mid-parent heterosis index surpassing 30%. However, the P1 × P3 combination displayed a positive advantage solely in seedling height, and even exhibited a hybrid disadvantage in fresh weight. Consequently, the F12 and F21 were determined to be superior hybrids, while F13 and F31 was deemed inferior hybrids. On the other hand, there was no significant correlation observed between the phenotype of F_1_ seedlings and contemporary hybrid seed weight, protein content, or fat content (**Figure 1D**). For instance, although the hybrid seeds of F13 had the highest values in 15-seed weight and seed fat content, their seedlings exhibited negative biomass heterosis. In addition, significant positive correlations were found among seedling first leaf length, first leaf width, seedling fresh weight, and root fresh weight (**Figure 1D**). Based on these observations, we hypothesize that the seedling dominance of F_1_ hybrids is not influenced by the inclusion content in the hybrid seed. Instead, it is linked to an increase in the photosynthetic area and the coordination between shoot and root.

### 3.2 Identification of differentially expressed genes between hybrids and their parents

RNA-Seq analysis was conducted on F_1_ hybrid and parental seedlings to investigate the underlying factors contributing to the notable trait differences observed in the two hybrid combinations. A total of 21 samples were generated, consisting of three biological replicates for each of the seven genotypes. All library produced valid sequencing data exceeding 5.9 Gb and Q30 values above 97% (**Supplementary Table S3**), and the Pearson’s correlation coefficients between the three replicates of the same genotype exceeded 0.8 (**Supplementary Figure S2A**). Additionally, the majority of reads in each sample were successfully aligned to the exon region of the reference genome with a unique mapping rate exceeding 90% (**Supplementary Figures S2B, C**). To validate the credible of the RNA-Seq data, qRT-PCR assays were performed on five randomly selected genes. The results demonstrated a strong significant positive correlation (Pearson coefficients, *p* < 2.2e-16) and consistent expression trends between the qRT-PCR expression levels and the RNA-Seq data (**Supplementary Figure S3**). Thus, the quality of the transcriptome data is sufficient for subsequent analysis.

**Figure 2:**
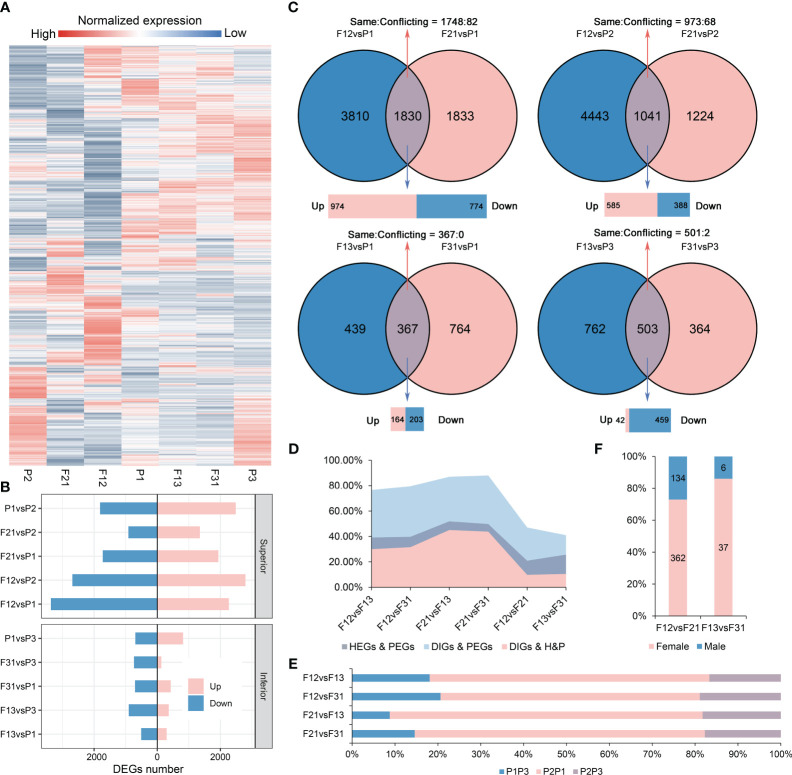
Gene expression profiles in all hybrids and their parents. **(A)** Gene relative expression levels of seven genotypes; **(B)** The DEGs numbers in two crossing combination; **(C)** Intersection analysis of DEGs between hybrids and parents within the same crossing combination; **(D)** Effect of parental gene expression differences on the expression differences of hybrids. HEGs, the DEGs between hybrids; PEGs, the parental DEGs, DIGs, the differential inheritance genes; H&P, the intersection genes of HEGs and PEGs; **(E)** Contribution of parental selectively inherited expression to DEGs among hybrids. P1P3, the DEGs between P1 and P3, in a similar fashion; **(F)** Contribution of parental selectively inherited expression to reciprocal hybrid DEGs.

A total of 30,756 genes were obtained through RNA-Seq analysis from the seven materials. As expected, all hybrids showed significant reprogramming in gene expression profiles compared to their parents (**Figure 2A**). Notably, the number of DEGs in the strong hybrids exceeded those in the weak hybrids, with average DEGs counts of 5562 for F12, 2964 for F21, 1036 for F13, and 999 for F31 when compared to their respective parents (**Figure 2B**, **Supplementary Figure S4**). Intersection analysis revealed that, in the hybrids, the common DEGs compared to parents (copDEGs) were primarily up-regulated in superior F_1_, while the opposite pattern was observed in inferior F_1_ (**Figure 2C**). Additionally, only a few genes of copDEGs exhibited conflicting directions in expression alternation between the reciprocal hybrids.

Strong hybrids exhibit a higher number of parental differentially expressed genes (PEGs) compared to weak hybrids (**Figure 2B**). Meanwhile, reciprocal hybrids generally do not share hybrid-parental DEGs (**Figure 2C**). To elucidate the role of parental DEGs in the formation of DEGs among hybrids, a comprehensive intersection analysis was performed (**Supplementary Figures S5A-F**). The results displayed that the overlap between hybrid DEGs (HEGs) and PEGs varied from 39.18% to 51.87%, depending on the hybrid combinations being compared (**Figure 2D**). A significant portion of these genes were differentially inherited genes (DIGs), indicating that the hybrids selected different expression levels from their parents, resulting in divergent expression levels in the hybrids. The DIGs exhibited a highly significant overlap with PEGs, ranging from 79.56% to 88.05%. This range also fell within the 30.02% to 45.11% range when compared to the list of DEGs/PEGs intersections (H&P). The DEGs between strong and weak hybrids were primarily comprised of DEGs derived from parent P2 and other parents. Notably, the DEGs from P2 and P1 accounted for more than 60% of these differences (**Figure 2E**).

In contrast, the percentage of H&P was reduced to 20.89% (F12/F21) and 25.67% (F13/F31) in reciprocal hybrids, and a similar trend was observed in the overlap between DIGs and PEGs, as well as between DIGs and H&P (**Figure 2D**). Most of the production of DIGs among reciprocal hybrids was found to be a result of selective inheritance from the female parent, although DIGs accounted for only approximately 6% of the HEGs (**Figure 2F**). Furthermore, the expression profiles of the parental DEG list indicated that the expression level of a specific parent was largely co-selected in reciprocal hybrids (**Supplementary Figures S5G, H**). Therefore, the DEGs between reciprocal hybrids are mostly independent of parent-of-origin or cytoplasmic effects, and parental DEGs play a significant role in the emergence of hybrid DEGs across different crossing combinations. Interestingly, these findings align with our previous studies on DNA methylation in contemporary immature hybrid seeds (Chen et al., 2022b).

### 3.3 Analysis of gene expression reprogramming patterns in F_1_ hybrids

**Figure 3:**
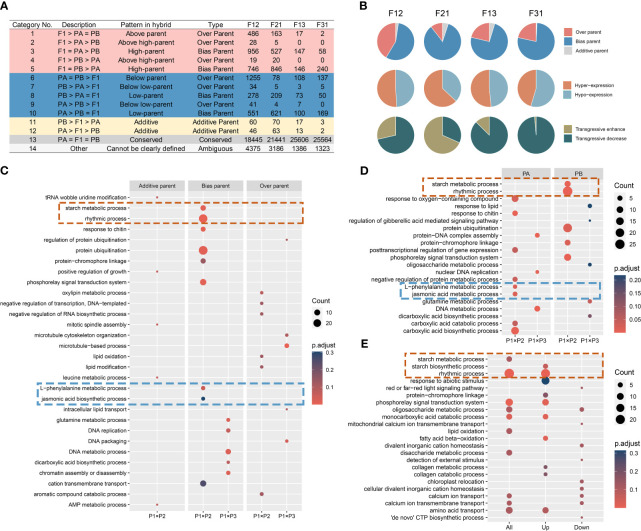
Reprogramming of gene expression in seedlings of all F_1_ hybrids. **(A)** Gene expression remodeling pattern of F_1_; **(B)** Reshaping patterns as a percentage of the non-additive expression genes; **(C-E)** GO enrichment analysis of gene sets. **(C)** The compiled consistent genes in reciprocal hybrids; **(D)** The shared DIGs of reciprocal hybrids; **(E)** The DEGs of crossing combinations.

The gene expression levels in the F_1_ hybrid can display additive, nonadditive, or conserved patterns compared to those of the parents. Additive gene expression levels fall between those of the two parents, while nonadditive gene expression levels indicate a bias towards or beyond a single parent. Conserved genes denote similar gene expression levels between the hybrid and their parents. Based on these definitions, previous studies on *Arabidopsis* biomass heterosis were referenced (Liu et al., 2021). All genes were classified into fourteen subcategories and five major categories (**Figure 3A**).

Overall, most genes in the F_1_ hybrids did not undergo expression recombination (Class 13), and the ones that did showed a preponderance of non-additive expression (Class 1-10), with only a minority displaying an additive pattern (Class 11-12). The number of non-additively expressed genes (NEGs) and additively expressed genes (AEGs) was higher in superior hybrids compared to inferior ones (**Figure 3A**). Secondly, in NEGs, bias parental expressed genes (BPGs) were the prevailing type, and hyper-expressed genes (Class 1-5) were approximately equivalent in percentage to the hypo-expressed genes (Class 6-10). A large number of the over parental expressed genes (OPGs) are transgressive decrease genes in the weak cross combinations, but with no distinguishable pattern in the strong crossing combinations (**Figure 3B**). Furthermore, it has been identified that the majority of the expression reprogramming genes have origin in regions that were differentially expressed in the parental transcriptomes (**Figure 3A**).

The direction of expression remodeling varied due to differences in gene expression between the reciprocal hybrids. Upon analyzing the compiled consistent genes in reciprocal hybrids (ReCGs), the results were similar to the reshaping patterns observed in individual F_1_ hybrids (**Supplementary Figure S6**, **Supplementary Table S4**). However, there were some differences. Firstly, the percentage of BPGs increased to over 80%, which means that the proportion of NEGs in regions of parental DEGs also increased. Additionally, in strong hybrid combinations, the number of hyper-expressed genes was approximately double that of the hypo-expressed genes (831 versus 420), whereas in weak combinations, the numbers of two type genes were similar (111 versus 124). Among the strong combinations, the number of positively OPGs exceeded the number of negatively OPGs (39 versus 23), while all OPGs shared by the inferior hybrids exhibited down-regulation of expression (48 in total).

To investigate the biological roles of the remodeling genes in the hybrids, GO enrichment analysis was performed on the three categories of ReCGs (**Supplementary Table S5**). In phenotypically advantage hybrids, AEGs were predominantly associated with mitosis, while BPGs were implicated in activities such as starch metabolism, circadian rhythms, signaling pathways, and the metabolism of L-phenylalanine and jasmonic acid. Furthermore, OPGs were found to be involved in RNA metabolism, lipid compound modification. In phenotypically disadvantage hybrids, the biological pathways enriched in both BPGs and OPGs were primarily related to cellular growth processes, including DNA metabolism and chromosomal organization (**Figure 3C**). Interestingly, when comparing these pathways with the enrichment results of shared DIGs within the hybrid combinations, it was observed that starch metabolism and circadian processes in the strong hybrids originated from P2, while L-phenylalanine and jasmonic acid metabolism were inherited from P1 (**Figure 3D**, **Supplementary Table S6**). Similarly, DNA metabolism-related processes in the weak hybrids came from P1, and glutamate and dicarboxylic acid metabolic pathways were inherited from P3. The GO enriched lists of DEGs in the P1 × P2 and P1 × P3 combinations revealed similar biological processes in the ReCGs and the DEGs between strong and weak hybrids. Notably, in the phenotypically advantageous combination, highly expressed genes associated with the circadian rhythm and starch synthesis pathways were significantly enriched (**Figure 3E**; **Supplementary Table S7**). All DEGs were categorized, and it was found that they primarily consisted of BPGs and a small proportion of OPGs in the P1 × P2 combination (**Supplementary Figure S7A**), which aligns with the findings from Section 3.2 (**Figure 2F**). Additionally, the enrichment analysis of gene expression remodeling in the all hybrids also revealed that the superior hybrids were involved in processes related to photosynthesis and photosynthetic organs (**Supplementary Figure S7B**, **SupplementaryTable S5**). The aforementioned findings lead to the hypothesis that non-additive expression, largely influenced by parental selective inheritance, is the key driving force in shaping expression profiles in soybean F_1_ hybrid seedlings. Furthermore, these genes display distinct parental complementarity in their biological functions, and the coordination of specific biological processes contributed by the parents in the hybrid offspring accounts for heterosis power differences.

### 3.4 The WGCNA of soybean seedling expression profiles

The method of DEGs analysis based on statistical thresholds does not allow us to determine whether there is any coordination of gene expression changes among or between taxa. To address this limitation, WGCNA utilizes the multiple expression data of all genes to identify sets of genes with highly coordinated changes. By calculating the correlation between these gene sets and phenotypes, this analysis approach helps uncover the molecular basis of phenotypic dominance in conjunction with gene remodeling patterns (Liu et al., 2021).

**Figure 4:**
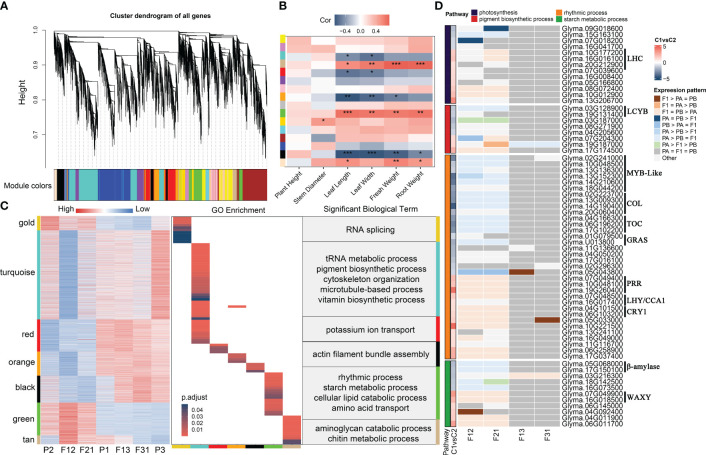
WGCNA of soybean seedling expression profiles and identification of key genes associated with phenotypic heterosis. **(A)** Expression module dendrogram; **(B)** Correlation of modules with traits. “***” indicates *p* < 0.001, “**” indicates *p* < 0.01, “*” indicates *p* < 0.05; **(C)** Expression heatmap and enrichment analysis of key modules; **(D)** Key genes for seedling heterosis. C1vsC2 refers to the mean value of expression levels for F12 and F21 compared to the mean value of expression levels for F13 and F31.

Using the parameters outlined in Section 2.5, the soft threshold for constructing the network was calculated using the 18934 genes obtained from filtering (**Supplementary Figure S8A**). The one-step method provided fifteen gene modules with high internal connections, while only 127 genes could not be generalized to any gene modules (**Figure 4A**, **Supplementary Figures S8B-E**). The aims of the current manuscript was to examine the correlation between gene networks and seedling heterosis. Hence, only seedling phenotypes were considered for the correlation analysis with gene modules. Eight modules showed significant associations with phenotypes (**Figures 4B**, **Supplementary Figure S9A**). However, in the bisque module, the correlation values between gene significance (GS) and module membership (MM) for leaf length, fresh weight and root weight were all less than 0.2 and *p* > 0.05 (**Supplementary Figures S9B-D**). For the remaining seven modules, the correlation between GS and MM varied between 0.23 and 0.78 depending on the phenotype. Additionally, the correlation’s significance was also significantly below 0.01 (**Supplementary Figure S10**). Of these, gold module displayed a positive correlation with stem thickness, while turquoise and red module exhibited a negative correlation with leaf length and width. Orange module, on the other hand, had negative correlations with leaf length, width, and seedling fresh weight. The modules of black, green, and tan were significantly correlated with leaf length, leaf width, fresh weight and root fresh weight, with all of them showing positive correlations except for black module.

Gene expression for seven genotypes was acquired in these modules. The results suggest that the gene expression patterns align with the correlation between modules and phenotypes. Specifically, negative correlations with traits were observed when the expression levels of genes within strong hybrids were lower compared to other samples, and vice versa (**Figure 4C**). Interestingly, with the exception of turquoise and black module, which showed an expression pattern unique to F12 and F21, gene expression in the remaining five modules was dominated by parental selective inheritance pattern. Additionally, the GO terms for these functional modules demonstrate little redundancy, with only orange and turquoise module have shared entries in cellular assembly and molecular function (**Figure 4C**). This finding indicates that the seven gene modules have distinct roles in shaping hybrid dominance for soybean seedling. Through analysis of the GO enrichment findings (**Figure 4C**; **Supplementary Table S8**), it has been determined that gold module is primarily associated with RNA splicing and sugar catabolism. turquoise module is linked to tRNA, quinone metabolism, pigment and vitamin synthesis, as well as cytoskeleton organization. Red module is primarily concerned with potassium ion transport, auxin response and protein glycosylation. Biological processes regulated by orange module did not show significant enrichment, with the most notable term being photosynthesis. Black module was involved in actin movement, mitotic negative regulation pathways and the gibberellin signaling pathway. Green module was associated with circadian regulation, starch metabolism, lipid degradation. Tan module was involved in stress-related processes, such as glucosamine catabolism and chitin metabolism. Furthermore, all seven modules demonstrated the presence of cell cycle and cell division-related processes, but none of them exhibited significant enrichment (**Supplementary Table S8**). In summary, the aggregation of optimal expression patterns of key biological processes within F_1_ hybrids is crucial for the emergence of seedling phenotypic superiority.

Photosynthesis (GO:0015979), pigment biosynthetic process (GO:0046148), rhythmic process (GO:0048511), and starch metabolism (GO:0005982) were consistently enriched in hybrid expression remodeling genes and key gene modules (**Figures 3C-E**, **4C**, **Supplementary Figure S7B**). This result is in line with previous studies on plant seedling heterosis (Thiemann et al., 2014; Liu et al., 2021; Hu et al., 2022), indicating the significance of these four pathways as key biological processes for phenotypic dominance in F_1_ seedlings. The unique genes in the four terms were collated based on GO annotations and screened for hub genes for seedling biomass heterosis according to the following criteria: (1) remodeling of gene expression occurs within at least one hybrid; (2) |gene expression level (E) _F12/_E_F21_| < 1; and (3) |E_P1 × P2_/E_P1 × P3_ | >1. These criteria resulted in the selection of 66 eligible candidate genes (**Figure 4D**; **Supplementary Table S9**). Among these, the majority of genes involved in photosynthesis exhibited high expression levels in the strong hybrids. However, for other processes, the number of genes with high and low expression in the two combinations was similar. Consistent with previous analyses, the expression levels of candidate genes within F12 and F21 were predominantly biased towards the P2 parent, while the majority of candidate genes in F13 and F31 displayed a conserved expression pattern. Notably, genes belonging to different gene families demonstrated contrasting expression trends between the two crossing combinations (**Figure 4D**). Genes encoding the light-harvesting complex (LHC) and the key *WAXY* gene for starch synthesis enzyme showed high expression in the superior hybrids, whereas genes encoding lycopene beta-cyclase (LCYB) and β-amylase exhibited high expression in the inferior hybrids. In circadian rhythm-related processes, *MYB-like*, *CONSTANS-like* (*COL*), *Timing of CAB expression* (*TOC*), and *Gibberellin-acid insensitive, repressor of gal-3 and scarecrow* (*GRAS*) genes displayed low expression in the superior hybrids, while *Pseudo-response regulators* (*PRR*), *Late elongated hypocotyl* (*LHY*)/*CCA1*, and *Cryptochrome 1*(*CRY1*) family genes exhibited high expression in the superior hybrids. Furthermore, these genes are also potential candidates within the QTL interval for soybean plant traits [the QTLs information from SoyBase (https://www.soybase.org/), (**Supplementary Table S10**)]. For instance, the LCYB gene *Glyma.19G131400* is a candidate gene for regulating leaf area (Orf et al., 1999), leaf length and width (Orf et al., 1999; Kim et al., 2005), which may also affect the biomass of soybean shoot (Nicolás et al., 2006). *Glyma.07G049400*, belong to *PRR* family, may play a role in regulating leaf area and chlorophyll sulfur content in leaves (Mansur et al., 1993; Li et al., 2010). Under low phosphorus conditions, *Glyma.15G163100* has an impact on the accumulation of root biomass (Zhang et al., 2016). It is suggested that changes in the amplitude of circadian rhythms, increased photosynthetic capacity and enhanced starch synthesis are responsible for biomass heterosis in soybean hybrid seedlings.

### 3.5 Effects of DNA methylation in immature seeds on seedling gene expression

**Figure 5:**
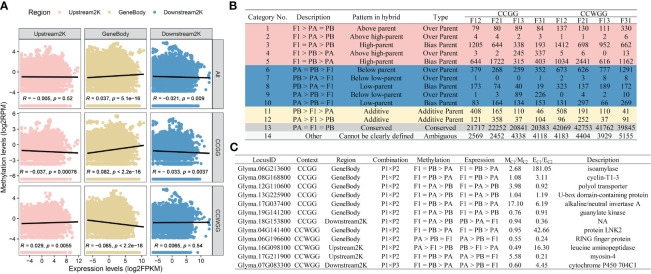
Effect of DNA methylation in contemporary hybrid seeds on gene expression in seedlings. **(A)** Correlation between methylation levels of gene regions and expression levels. **(B)** Classification of DMSs loci within F_1_ hybrids. **(C)** Overlapping genes for remodeling patterns shared in hybrid combinations.

Previous studies in rice have demonstrated that DNA methylation in hybrid contemporary seeds or embryos can affect the expression levels of hybrid seedlings (Zhou et al., 2021; Liu et al., 2023). We speculate that a similar situation exists in soybean hybrids. To investigate this, data from our previous studies (Chen et al., 2022b) were collected and to examine the relationship between methylation levels of hybrid contemporary seeds and expression levels of F_1_ seedling. The correlation analysis revealed variation in the correlation between the level of expression and DNA methylation based on gene region and methylation context (**Figure 5A**). The gene expression levels were positively correlated with the overall or CCGG sites methylation levels of the gene body, while it’s showed a negative correlation with the CCWGG sites. However, the status of upstream 2000 bp region was the opposite of the gene body, and the total methylation levels are not related to the level of gene expression. The methylation levels of downstream 2000 bp region exhibited a strong negative correlation with gene transcript abundance only in global or CCGG sites. Furthermore, the differentially methylated sites (DMSs) were categorized according to the gene expression recompilation pattern in F_1_ seedlings, resulting in four outcomes (**Figure 5B**, **Supplementary Figures S11A, C**): (1) Conserved sites predominated the hybrids, regardless of their methylation background, with similar to the seedling expression recompilation. The DMSs sites were mainly dominated by non-additive methylation sites (NMSs), and additive methylation sites (AMSs) constituted approximately 3-17% of the total depending on the hybrids; (2) The inferior hybrids exhibited a higher proportion of over parent methylation sites (OMSs) and hypomethylated sites in comparison to the superior hybrids. Meanwhile, F12 and F21 are dominated by biased parent methylation sites (BMSs), in contrast to F13 and F31. Only F13 had a lower proportion of BMSs than OMSs in the CCWGG context; (3) The NMSs in various hybrids and cytosine context were primarily derived from the parental methylation difference region (Class 2-5 and 7-10). In the CCGG context, approximately 15% of NMSs were derived from the parental conserved region (Class 1, 6) in P1 × P2, increasing to 24% in P1 × P3. Conversely, in the CCWGG context, the figures were 20% in P1 × P2 and 37% in P1 × P3; (4) Our previous analysis indicated that DMSs in hybrids were more concentrated within gene regions than the natural distribution of methylation sites (Chen et al., 2022b). Similar results were found for the NMSs and AMSs within hybrids, with the distribution of sites increasing from 13.45% to 21.54-38.82% in CCGG type and from 5.68% to 6.81-12.92% in CCWGG site, depending on the genotype (**Supplementary Figures S11B, D**).

Following this, the NMSs/AMSs were utilized to match with the NEGs/AEGs. It is worth noting that a gene could have several methylation sites, and whether the status consistency or conflict of these sites was not considered during matching. Rather, the focus was on whether they align or deviate with the corresponding gene’s remodeling pattern. The findings reveal identification of approximately 5,500 overlapping genes at CCGG sites and roughly 3,000 at CCWGG sites (**Supplementary Figure S12**; **Supplementary Table S11**). The majority of genes that exhibit DNA methylation consistent with the expression reprogramming pattern belong to conserved types (Class 13) and other types (Class 14), with only a small number being non-additive or additive. Additionally, certain genes exhibit contrasting pattern in the two remodeling modes (e.g., belonging to F1 > PA > PB in DNA methylation and PB > PA > F1 in gene expression). Proportionally, the proportion of genes displaying consistent or contradictory types in CCGG was only 0.3%, which is lower than the 0.6% observed in CCWGG. The selectively inheritance pattern contributed to more than 95% of concordant genes, regardless of the type of methylation. There were very few genes exhibiting hypomethylation with the hyper-parental type (PA = PB > F1). In addition, the 15 genes were selected exhibited either consistent or inconsistent patterns in reciprocal hybrids, including 7 in CCGG and 5 in CCWGG (**Figure 5C**). These genes that displayed noticeable differences in mean methylation levels among hybrid combinations also showed significant variation in the average expression levels of seedlings. Interestingly, the functional annotation of these genes suggests their involvement in cytokinesis, such as cyclin-T1-3, and starch synthesis pathways, such as isoamylase. Among these, the gene *Glyma.17G037400*, which is a key component of the hybrid seedling expression network, was also found to the overlapping gene of remodeling pattern. It should be noted that four overlapping genes are also candidate genes for soybean trait QTL (**Supplementary Table S10**). In particular, *Glyma.19G141200* controls not only the length, width, and area of leaves but also the dry weight of the shoot (Orf et al., 1999; Kim et al., 2005; Nicolás et al., 2006). The results suggested that DNA methylation remodeling in contemporary hybrid seeds may regulate gene expression in subsequent developmental stages, playing a role in the generation of seedling heterosis.

## 4 Discussion

### 4.1 Phenotypic dominance of soybean F_1_ seedlings

Previous research has demonstrated that F_1_ hybrid seedlings of plants generally display vigorous growth or increased biomass (Kwan et al., 2016; Sinha et al., 2020; Liu et al., 2021). Our research on soybeans revealed that F_1_ seedlings of P1 × P2 combination conferred a notable edge in leaf area and biomass (**Figure 1C**). Meanwhile, crossing may result in a phenotypical disadvantage or no heterosis (F13 and F31), as can be seen in oilseed rape (Hu et al., 2022). It is worth noting that, with regard to kinship, P2 (Y3) is a germplasm from Italy, whereas P1 (Jilin 38) and P3 (Jilin 47) are cultivars developed in Jilin Province. Hence, P1 may be more closely kinship to P3 than to P2. It has been suggested that the greater the genetic distance between crossing parents, the more significant the advantage in phenotype of their hybrid offspring (Wang et al., 2023). Previous studies have repeatedly demonstrated a correlation between biomass and genuine leaf area in hybrid seedlings (de Leon et al., 2001; Kwan et al., 2016; Mei et al., 2017; Li et al., 2021a). Our study exhibit that the length and width of the first leaf had a noteworthy positive correlation with plant fresh weight (**Figure 1C**). Furthermore, if the hybrids lacked a positive advantage in leaf traits, there was no positive benefit in all of the seedling phenotypes except for plant height (**Figures 1B, C**). Greater leaf size of F_1_ seedling has provided it with a larger photosynthetic area, which consequently promotes nutrient accumulation for growth and development. Strong seedlings can create a foundation for future seed yield, possibly explaining why two hybrid combinations exhibit the same trend in yield advantages (Wang et al., 2018; Song et al., 2020). Furthermore, Liu et al. (Liu et al., 2021) discovered that the growth advantage of hybrid seedling cotyledons was significantly greater and occurred earlier than the dominance of true leaves. This suggests a potential link between seed grain inclusions and the dominance of hybrid seedlings, as seed grain inclusions serve as an energy source for early seedling growth (Dornbos and Mullen, 1991; Kim et al., 2011). However, the correlation analysis between the weight, protein, and fat content of seed in contemporary soybean hybrids and their parents, as well as the seedling phenotypes, showed no significant correlation with seed phenotypes (**Figure 1C**). Similar findings have been observed in studies conducted with *Arabidopsis* and maize, and proposing that a rational allocation of metabolites by hybrid seedlings contributes to their growth advantages (Meyer et al., 2012; Kwan et al., 2016). Therefore, we have determined that the heterosis of biomass and leaf size in soybean are a result of seedling own growth and development, rather than being connected to the inclusion content of hybrid contemporary seeds. However, further verification is necessary to confirm this hypothesis.

### 4.2 Gene expression remolding pattern and key biological processes of biomass in seedling hybrids

Consistent with previous studies (Li et al., 2009; Chen et al., 2018), soybean F_1_ seedlings display a reprogramming event in gene expression compared to their parents (**Figures 2**, **3**). Moreover, more than 96% of DEGs demonstrate non-additive expression in hybrids seedlings, with about 73% showing biased parent expression. The number and proportion of DEGs and non-additive genes are higher in superior hybrids compared to inferior hybrids (**Figures 3A, B**). These results suggest that non-additive gene expression plays a critical role in the development of hybrid vigor in soybean seedling leaves and biomass, with biased parent gene expression being prominent among non-additive expression genes (Shahzad et al., 2020; Xiong et al., 2022). It is important to note that although the quantitative increase in the number of NEGs implies a soar of the gap in the parent-hybrid expression network, differences in the expression network do not necessarily result in a phenotypic advantage for the hybrids, as inferior hybrids also have many NEGs or AEGs (**Figure 3A**). Using enrichment analyses of these genes, we found that several sets of highly linked genes within superior hybrids tended to regulate different key biological processes (**Figures 3C-E**, **4C**), suggesting that phenotypic advantage in F_1_ requires the aggregation of optimal gene networks, in particular the complementary favorable expression patterns from bi-parents, rather than mere transcriptional remodeling. In addition, AEGs in strong hybrids are involved in cell division, AMP and amino acid metabolism (**Figure 3C**). They potentially help balance the expression or overexpression of biparental deleterious genes (Thiemann et al., 2014). The expression modules specific to hybrids mined in WGCNA also regulate crucial biological processes such as tRNA metabolism and cytoskeleton (**Figure 4B**). Thus, soybean seedling heterosis should rely on a mixed and coordinated expression regulatory network dominated by dominant effects and complemented by additive and hyper-parental effects. This has been repeatedly demonstrated in *Arabidopsis*, rice and maize (Song et al., 2013; Li et al., 2021b; Liu et al., 2021). Another noteworthy point is that most of the inherited expression in the strong and weak combinations came from non-common parents (P2 or P3), suggesting that the expression network of P1 is unfavorable for the heterosis at DAS15 (**Figure 3A**, **Supplementary Figures S5G, H**), and the mechanism may be related to selective silencing of the deleterious allele (Botet and Keurentjes, 2020). The dynamics of the 3D structure of chromosomes in oilseed rape F_1_ hybrids indicated that the activation of chromatin compartments is due to genetic distance between parents, leading to dominant expression (Hu et al., 2022). In this study, the expression disparities between bi-parents were proportionate to the differences between parents and hybrids (**Figure 2B**, **Supplementary Figure S4**). Moreover, it was observed that more genes depicted upregulated expression in strong hybrids compared to parents, whereas the opposite was observed in the weak crossing combinations (**Figure 2C**). These findings suggest a comparable mechanism at play in soybean.

Heterosis in plant leaves or biomass has been associated with circadian rhythms, photosynthesis, and starch synthesis (Ni et al., 2009; Groszmann et al., 2014; Kwan et al., 2016; Sinha et al., 2020; Li et al., 2021a). Rhythm factors, in particular, are recognized as important genes that regulate the overall expression network in plants (Chen, 2013). Previous research has demonstrated that *AtCCA1* exhibits a lower diurnal cycle amplitude in hybrids compared to parents and directly promotes starch accumulation (Ni et al., 2009; Groszmann et al., 2014). Furthermore, ChIP-Seq analysis supports the notion that *ZmCCA1* influences growth vigor by regulating energy metabolism pathways such as photosynthesis and ATP synthesis (Kwan et al., 2016). In this study, circadian rhythms were not only significantly enriched by multiple gene sets (**Figures 3C-E**, **4C**), but also showed expression differences in strong and weak hybrids (**Figure 4D**). The superior hybrids displayed low expression levels of *MYB-like*, *COL*, *TOC*, and *GRAS* gene families, while *PRR*, *LHY/CCA1*, and *CRY1* families showed the opposite pattern. These results differ from previous studies conducted in *Arabidopsis* and maize. For instance, *AtLHY* or *AtCCA1* exhibited expression levels that are biased towards low-value parent or surpass bi-parents, whereas *AtTOC1* showed a reverse trend (Groszmann et al., 2014). Similarly, *ZmCCA1* surpassed biparental expression only at night and remained comparable to the expression level of the higher-value parent during other times (Kwan et al., 2016). We propose that two factors may have contributed to these observations. Firstly, as a dicotyledonous plant, soybean may exhibit a distinct pattern of heterosis compared to monocotyledonous plants. Secondly, the samples were collected at 3:00 pm, a time when the circadian rhythm is likely to undergo transition. Additionally, circadian factors have been linked to the regulation of soybean growth stages. A notable example is *Glyma.07G048500* from the *LHY* family, which exhibits high expression levels in strong combinations and has been selectively favored during domestication to promote soybean flowering and maturation (Lu et al., 2020). Moreover, previous work reported a quadruple mutant of *GmLHY*s involving this gene, resulting in reduced plant height and internode length due to its regulatory mechanism associated with gibberellins (Cheng et al., 2019). *Glyma.07G048500* interacts with *Glyma.12G073900* from the *PPR* gene family, displaying functional antagonism (Li et al., 2020a). Both genes have resembling expression patterns in hybrids (**Supplementary Table S7**), indicating potential differences in rhythm amplitude and genes interaction between hybrid and inbred lines in soybean. The consistent presence of rhythmic cycle throughout growth and development may shed light on why hybrids demonstrate superiority in various traits at different growth stages. Moving forward, it would be beneficial to examine amplitude changes in diverse rhythmic genes and explore their distinct roles at different life stages. In addition, the *LHC* family genes in F12 and F21, *Glyma.10G012900* (Calvin cycle protein) and *Glyma.13G206700* (Glucose-6-phosphate translocator) had high peak expression, indicating higher photosynthetic activity (Bang et al., 2008) and carbon flux turnover rate (Wedel et al., 1997; Tamoi et al., 2005) than other genotypes (**Figure 4D**). Similarly, the inhibition of *WAXY* family and *Glyma.04G011900* (Glucose-1-phosphate adenylyltransferase) expression in genotypes with low biomass, alongside the activation of β-amylase, which are linked to starch or sugar content, also suggest the starch accumulation of the strong hybrids (Zhou et al., 2016; Chen et al., 2022a). These scenarios demonstrate the relationship between photosynthetic products and starch synthesis (Simkin et al., 2015) and partially endorse the hypothesis that the carbon metabolism network is optimized in dominant hybrids, providing more advantageous substances for plant growth and development to uphold phenotypic dominance of seedlings (Li et al., 2020b; Zhu et al., 2020; Li et al., 2021a; Xiong et al., 2022). Meanwhile, 34 out of 66 genes are candidate genes for QTL of leaf length, width, and area (**Supplementary Table S10**). Some of these genes may also regulate the accumulation of dry and fresh weight in leaves, shoots, and roots. Further exploration is needed to reveal their relationship with seedling heterosis in soybean.

Finally, one noteworthy matter is that the transcript levels of numerous genes in the superior hybrid at DAS15 were comparable to those of P2, implying a substantial influence of this parent in the formation of heterosis (**Figure 3A**). However, the proportion of inherited genes for expression levels between the two parents varied significantly in time and tissue specificity over total growth stages (Zhou et al., 2019; Liu et al., 2021). Therefore, to enhance the comprehension of the molecular mechanisms of phenotypic dominance in soybean hybrids seedling, it is necessary to conduct multiple successive studies with temporal correlation.

### 4.3 DNA methylation in contemporary hybrid seeds in relation to F_1_ seedling expression and heterosis

Our previous study revealed that DNA methylation remodeling genes in contemporary hybrid soybean seeds are associated with circadian rhythms, photosynthesis, and starch metabolism (Chen et al., 2022b). The significant correlation between methylation levels in gene regions and seedling expression levels, along with the similarity of reprogramming patterns (**Figures 5A, B**, **Supplementary Figures S11A, C**), which typically occur in plant hybrids of the same growth period (Orantes-Bonilla et al., 2023; Rao et al., 2023). It has been observed that DNA methylation patterns in soybean seed development can be partially inherited during the early stages of seedling growth (Lin et al., 2017), and research on rice F_1_ zygote has shown that DNA methylation is transmitted through cell division following reprogramming completion and is linked to histone modification in seedlings (Liu et al., 2023). Additionally, the DNA methylation status of rice hybrid shoots was discovered to persist during the emergence of young spikes, indicating lasting methylation at specific loci (Ma et al., 2021). The NMSs and AMSs, identified in combination with our study’s findings, were found to be enriched in gene regions (**Supplementary Figures S11B, D**), although few genes demonstrated consistent or paradoxical remodeling patterns in DNA methylation and gene expression (**Figure 5C**; **Supplementary Table S11**). Four of these few genes, *Glyma.06G196600*, *Glyma.06G213600*, *Glyma.08G168800*, and *Glyma.19G141200*, appear in the QTL candidate interval for soybean leaf traits and organ biomass (**Supplementary Table S10**). Furthermore, there is evidence indicating that CG and CHG methylation levels remain stable during soybean seed development (An et al., 2017), and important genes required for seed growth are not affected by non-CG demethylation (Lin et al., 2017). Therefore, it can be inferred that if the DNA methylation profiles formed during grain development is not entirely related to embryonic development, it must play a role at one or more stages of subsequent life process (Chen et al., 2022b). For example, promoting the formation of yield heterosis by influencing gene expression (Fu et al., 2023; Rao et al., 2023). This may also explain why AMSs and NMSs tend to be more distributed in gene regions than the methylated conserved sites (**Supplementary Figure S11**). On the other hand, certain genes in core heterosis biological process have been confirmed to play important roles in crucial biological mechanisms. For example, *Glyma.17G037400*, which is expressed positively in robust hybrids, encodes an alkaline/neutral invertase involved in sugar metabolism. The expression of this gene in soybean roots can be inhibited by metal stress (Han et al., 2023). A deletion mutation in the homologue *AT3G06500* can lead to stunted seedling growth, but this effect can be mitigated by externally administering gibberellin, and abscisic acid can induce a mutant-like phenotype in wild-type seedlings (Martín et al., 2012). Notably, the expression of this gene is regulated not only by gene factors that control circadian rhythms (Li et al., 2011), but also is a key gene in the advanced hybrid expression network (**Figure 4D**). Additionally, *Glyma.17G211900* encodes myosin-4 and exhibits contrasting expression patterns compared to *Glyma.17G037400*. A loss-of-function mutant of myosin-4 in *Arabidopsis* shows fewer starch grains and larger granules, but it does not affect total starch content (Vandromme et al., 2018). *Glyma.08G168800* encodes cyclin-T1-3 and its suppression of expression leads to dwarfing in wheat (Sharaf et al., 2023) and controls grain size in rice (Qi et al., 2012). This evidence suggests that DNA methylation in immature plant embryos is related to specific traits dominance for subsequent growth periods (Zhou et al., 2021). Hence, our hypothesis suggests that a portion of the DNA methylation state in hybrid seeds, may be transmitted to a specific growth phase through cell division, could control the transcriptional activity of targeted genes through its compiled functions, ultimately leading to the emergence of heterosis.

## 5 Conclusions

This research has revealed that F_1_ seedlings at the DAS15 stage exhibit significant growth advantages, resulting in larger leaf size and biomass compared to the parental plants. Significant variations in phenotypic dominance were observed among different crossing combinations. Furthermore, an analysis of the transcriptome profiles of seedlings and DNA methylation data from previous studies was conducted. The results revealed that the gene expression patterns in F_1_ seedlings are primarily influenced by non-additive remodeling, with parental dominant effects being the main component. The transcriptional profiles of the strong hybrids exhibited distinct characteristics, including an increase in gene expression reprogramming scale and an up-regulation DEGs numbers compared to the parental plants. Reshaping expression genes related to circadian rhythms, photosynthesis, and starch synthesis processes have been found to play a crucial role in facilitating robust seedling growth in superior hybrids compared to other genotypes. Additionally, the resemblance pattern between DNA methylation in contemporary hybrid seeds and gene expression in hybrid seedling implies that DNA methylation in immature seeds may play a role in regulating gene expression, and leading to the formation of seedling heterosis in soybean. Our study will contribute to a better understanding of plant hybrid dominance and the breeding of superior soybean cultivars.

## Data availability statement

The data presented in the study are deposited in the National Genomics Data Center (https://ngdc.cncb.ac.cn/), accession number PRJCA010543.

## Author contributions

XR: Writing – original draft, Writing – review & editing, Conceptualization. LC: Writing – original draft, Writing – review & editing, Formal analysis, Visualization. LD: Investigation. QZ: Investigation. DY: Investigation. XL: Validation. WC: Validation. ZZ: Resources. DZ: Resources. MZ: Resources. SY: Supervision, Funding acquisition. JZ: Supervision, Funding acquisition, Project administration.
