# Supplementary material for: Comparative transcriptomic analysis reveals the molecular mechanism underlying seedling heterosis and its relationship with hybrid contemporary seeds DNA methylation in soybean
Source: Front Plant Sci. 2024 Feb 19;15:1364284. doi: 10.3389/fpls.2024.1364284 (PMC10913200; doi:10.3389/fpls.2024.1364284)
Supplement: Supplementary file 1 [file DataSheet_1.pdf]

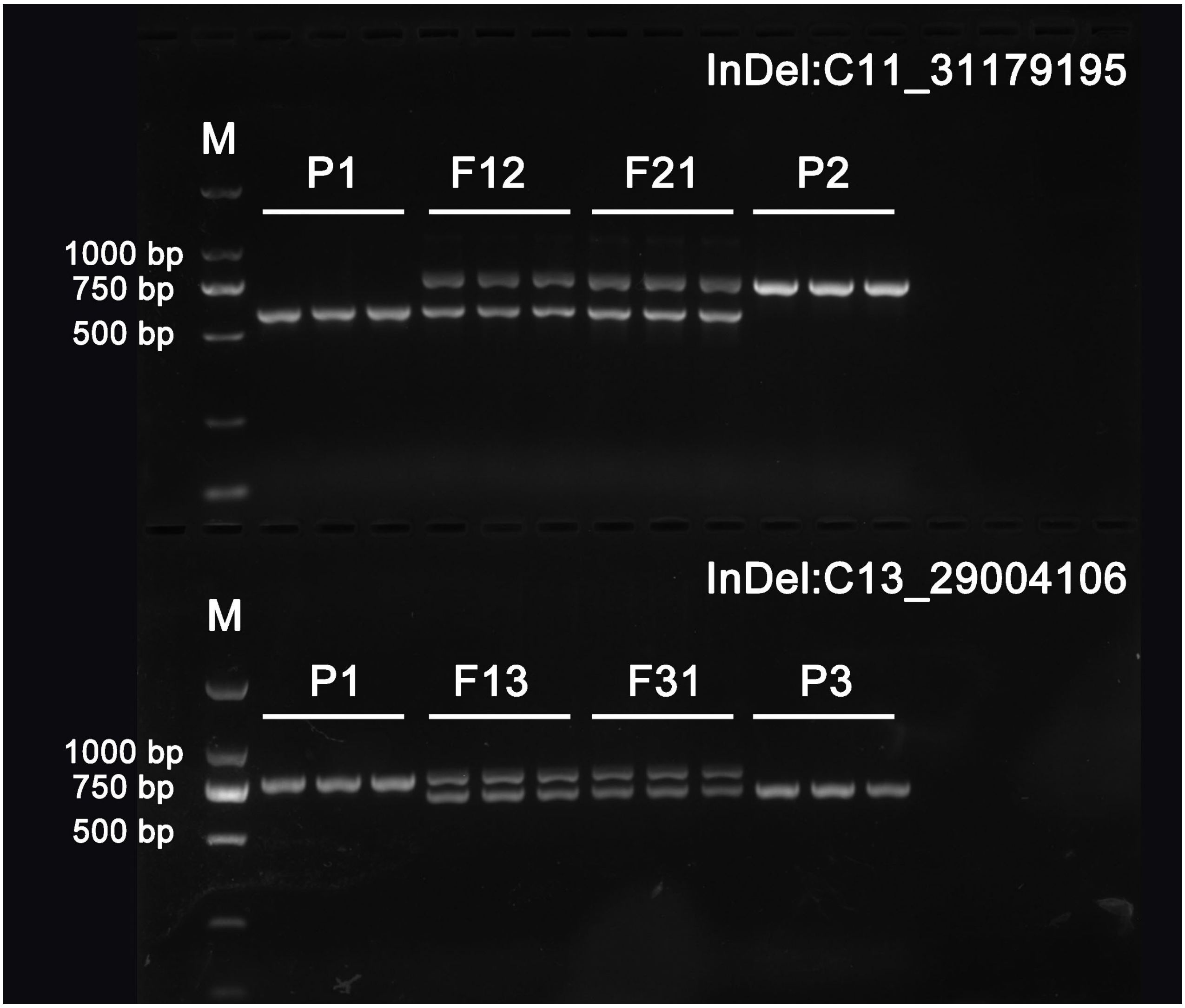

Figure S1 Identification of F_1 hybrid seedling using InDel marker

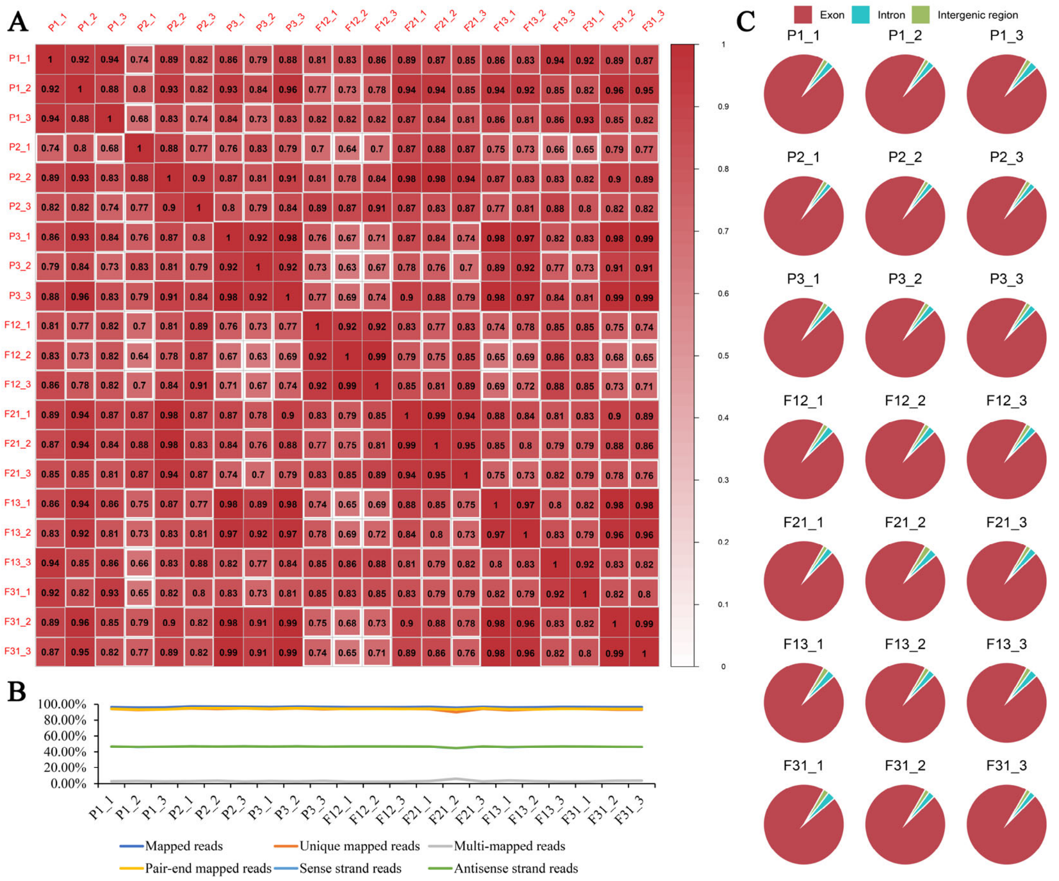

Figure S2 RNA-Seq data of seven genotypes

(A) Expression correlation analysis between three biological repeats; (B) Alignment quality of sample sequencing data on reference genomes; (C) Distribution of sample sequencing reads across gene regions.

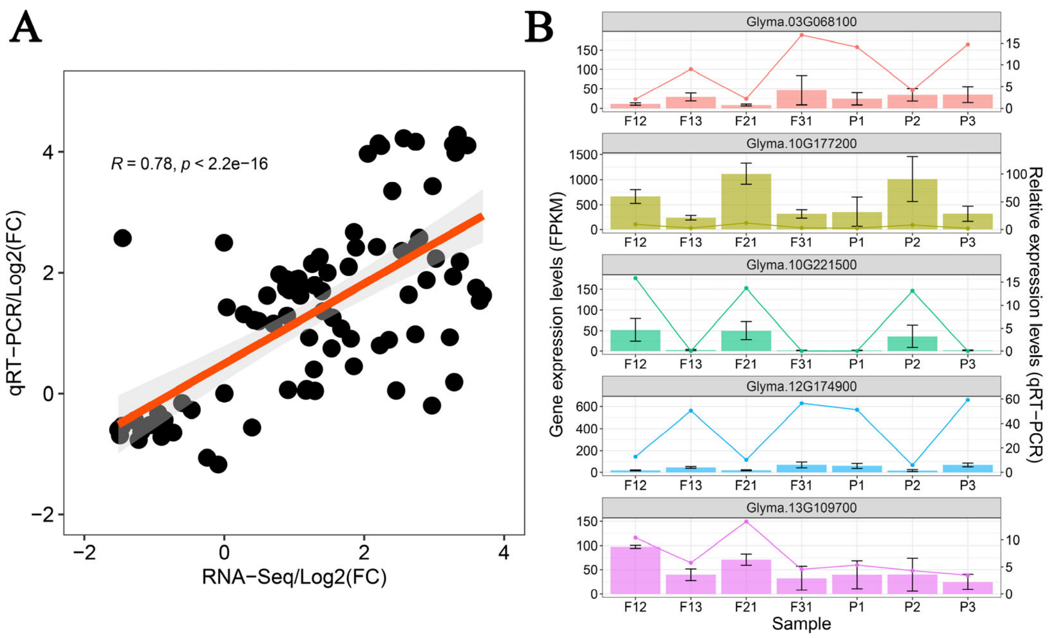

Figure S3 Correlation analysis between qRT-PCR and RNA-Seq

(A) Analysis of Pearson's correlation coefficient; (B) Consistent expression trends across genotypes in qRT-PCR and transcriptome. Dot plot is qRT-PCR data and bar plot is RNA-Seq data.

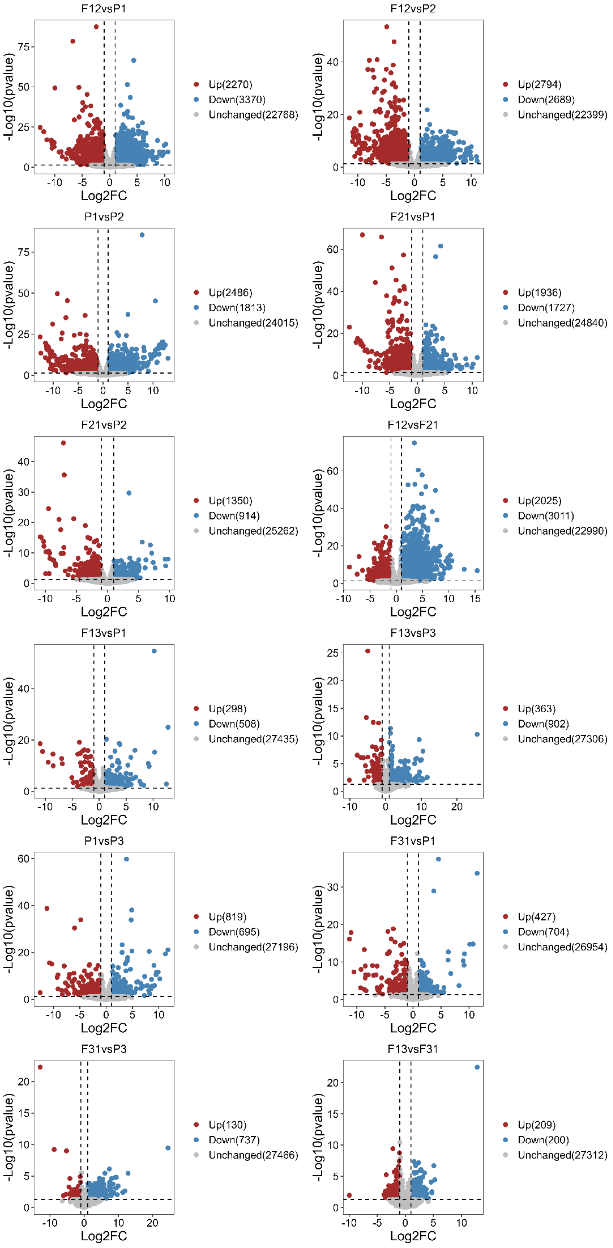

Figure S4 Differentially expressed genes analysis of soybean seedlings

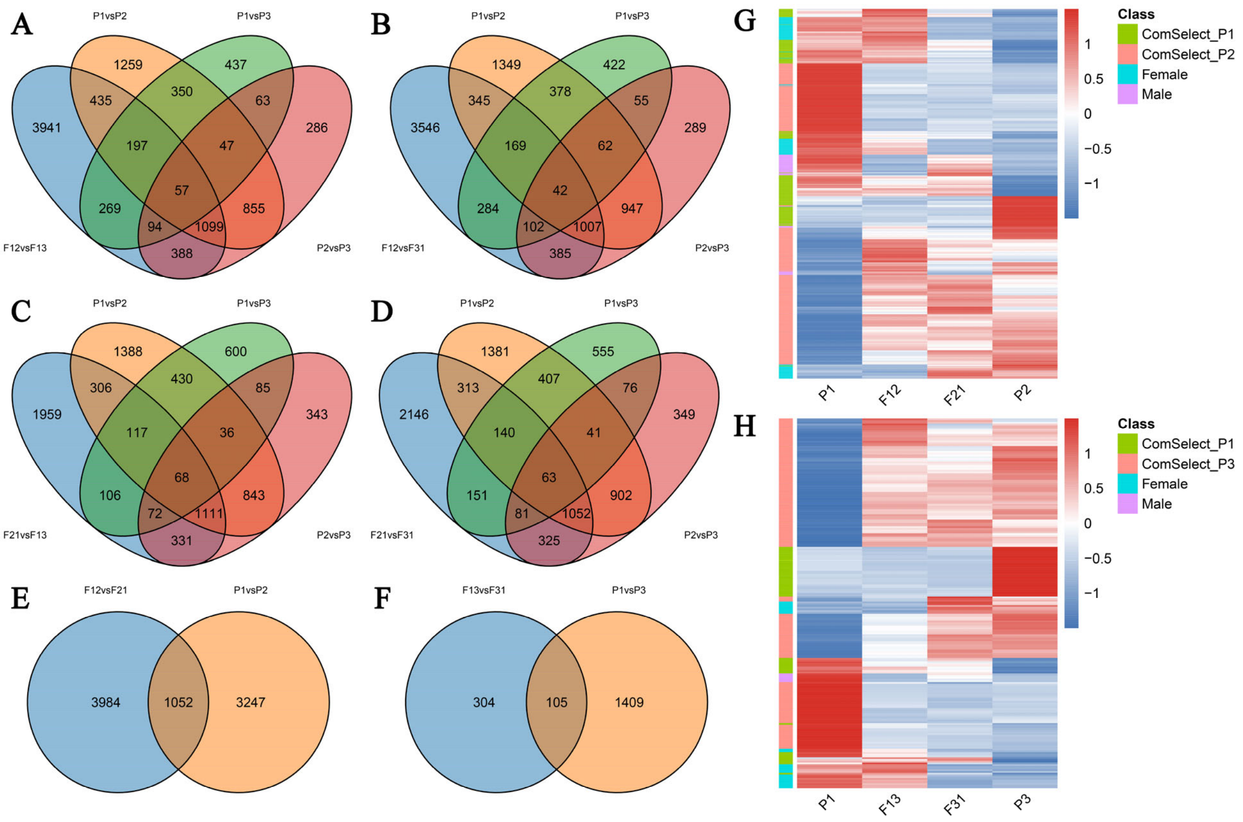

Figure S5 Impact of expression variations among inbred lines on expression variations among hybrids

(A-F) Intercross analyses of parental DEGs and hybrids DEGs; (G, H) Selective inheritance of parental expression levels by reciprocal hybrids. "ComSelect" indicates that the reciprocal hybrid inherits the same parent's gene expression levels.

Category No.	Description	Pattern in hybrid	Type	P1×P2	P1×P3
1	F1 > PA = PB	Above parent	Over Parent	33	0
2	F1 > PA > PB	Above high-parent	Over Parent	1	0
3	F1 = PA > PB	High-parent	Bias Parent	263	3
4	F1 > PB > PA	Above high-parent	Over Parent	5	0
5	F1 = PB > PA	High-parent	Bias Parent	529	108
6	PA = PB > F1	Below parent	Over Parent	20	47
7	PB > PA > F1	Below low-parent	Over Parent	2	1
8	PB > PA = F1	Low-parent	Bias Parent	116	34
9	PA > PB > F1	Below low-parent	Over Parent	1	0
10	PA > PB = F1	Low-parent	Bias Parent	281	42
11	PB > F1 > PA	Additive	Additive Parent	35	1
12	PA > F1 > PB	Additive	Additive Parent	14	1

Figure S6 Consistent reshaping pattern gene of the reciprocal hybrids

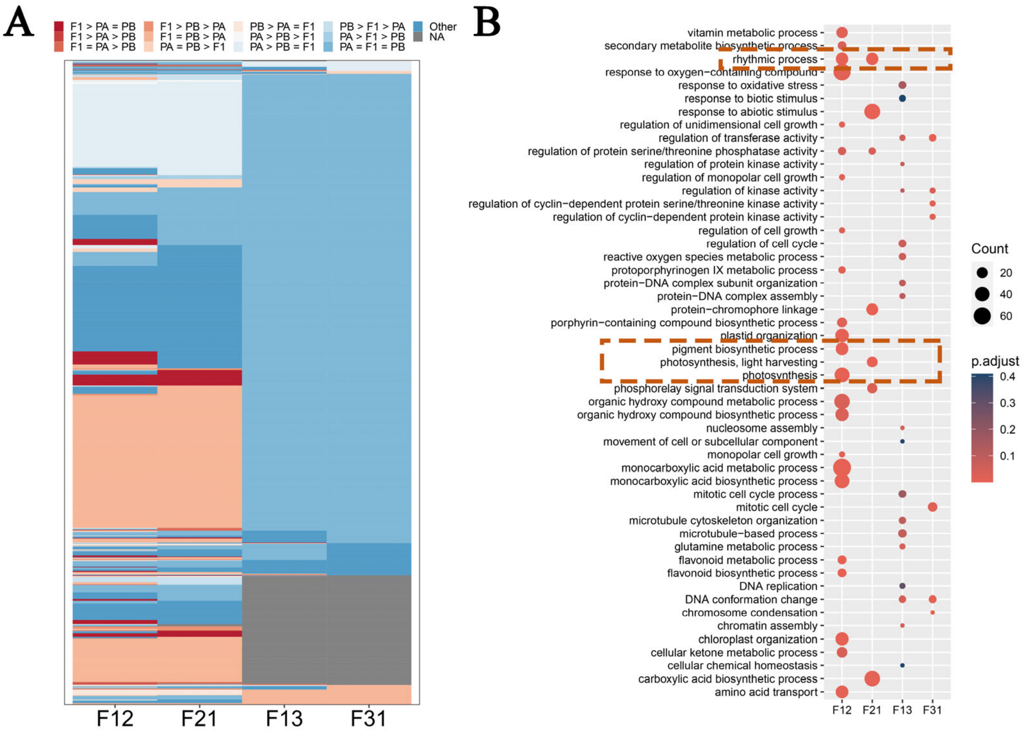

Figure S7 Distribution of expression remodeling patterns among hybrid combinations of DEGs (A) and GO enrichment analysis of remodeled expression genes (B)

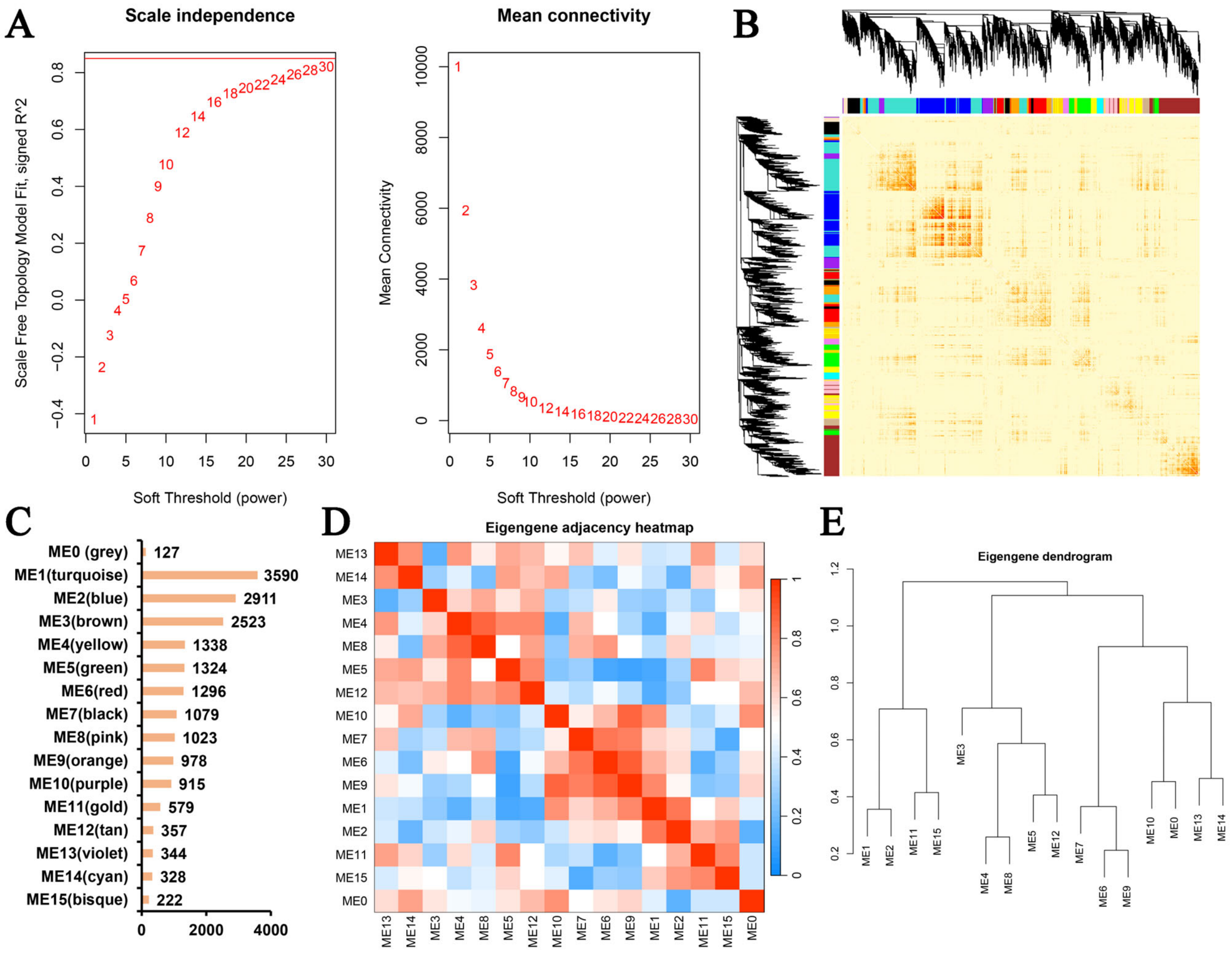

Figure S8 WGCNA of soybean seedling expression profiles

(A) Selection of soft thresholds; (B) Topological overlap matrix; (C) Number of genes within modules. ME stands for module; (D) Cluster analysis of eigenvalues between modules; (E) Correlation analysis of eigenvalues between modules.

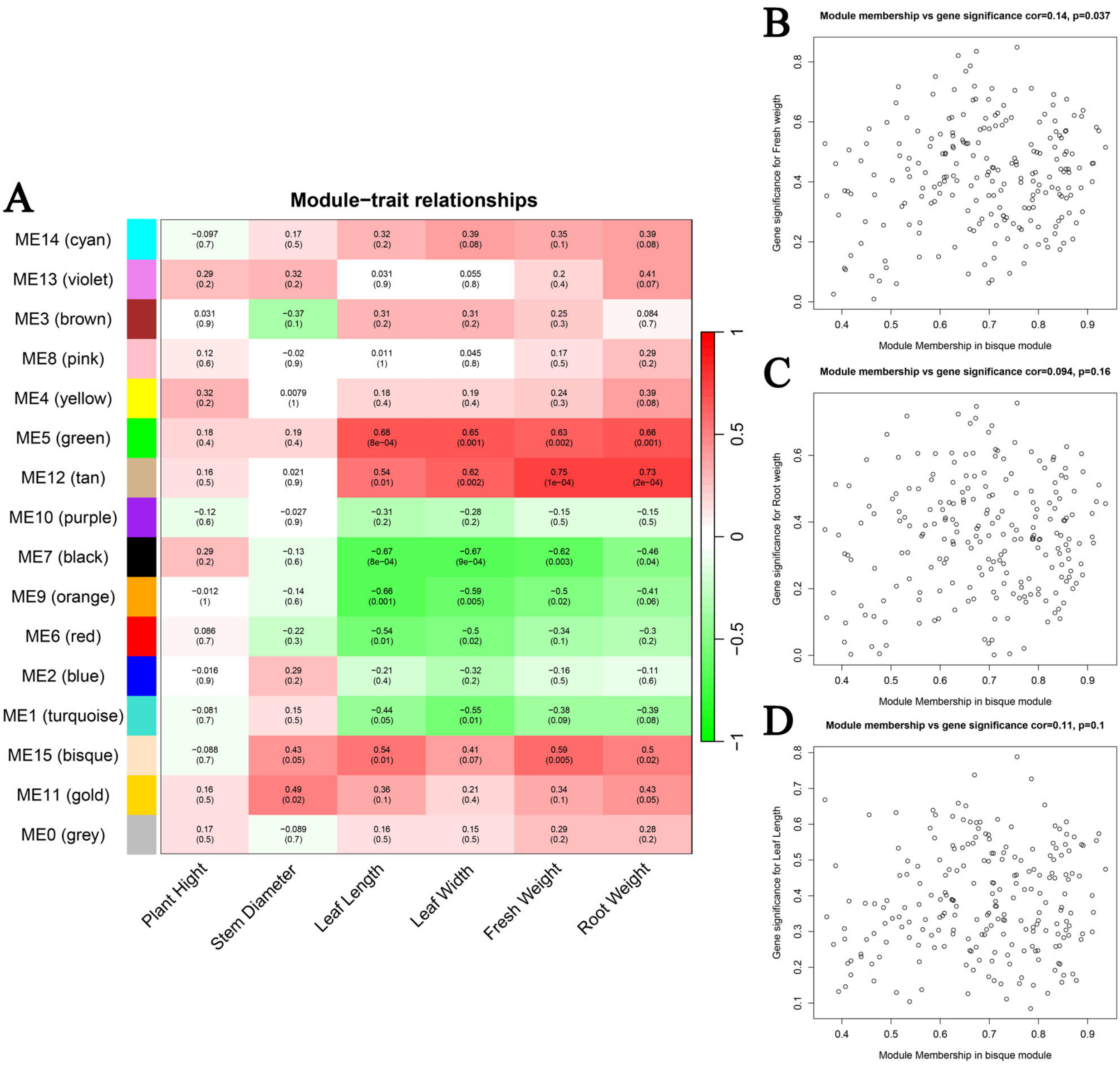

Figure S9 Soybean seedling co-expression gene modules and their relationship to phenotype

(A) Correlation of modules with traits; (B-D) Correlation of GS with MM in the bisque module.

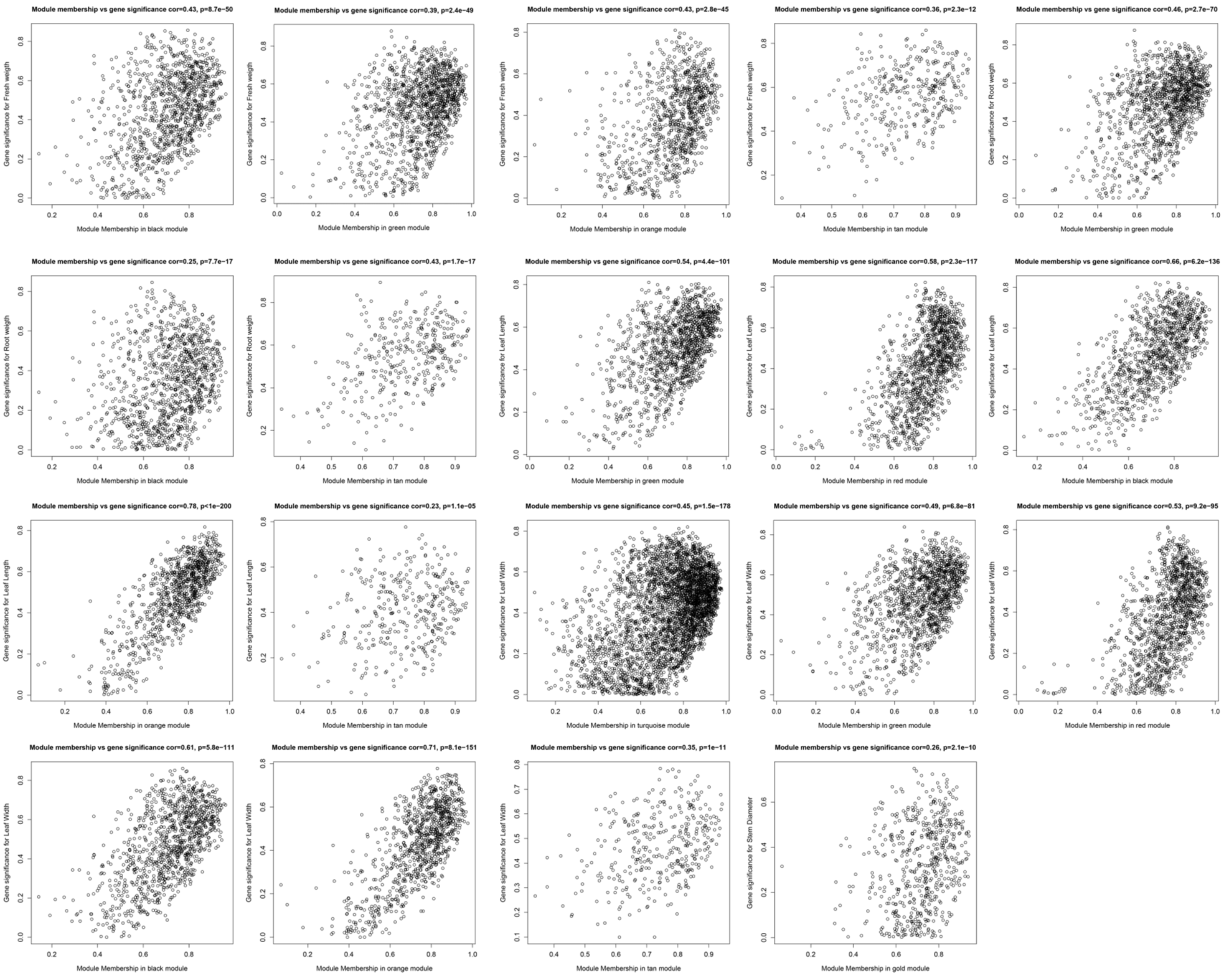

Figure S10 Correlation between module membership and gene significance in key expression modules of seedling phenotypes

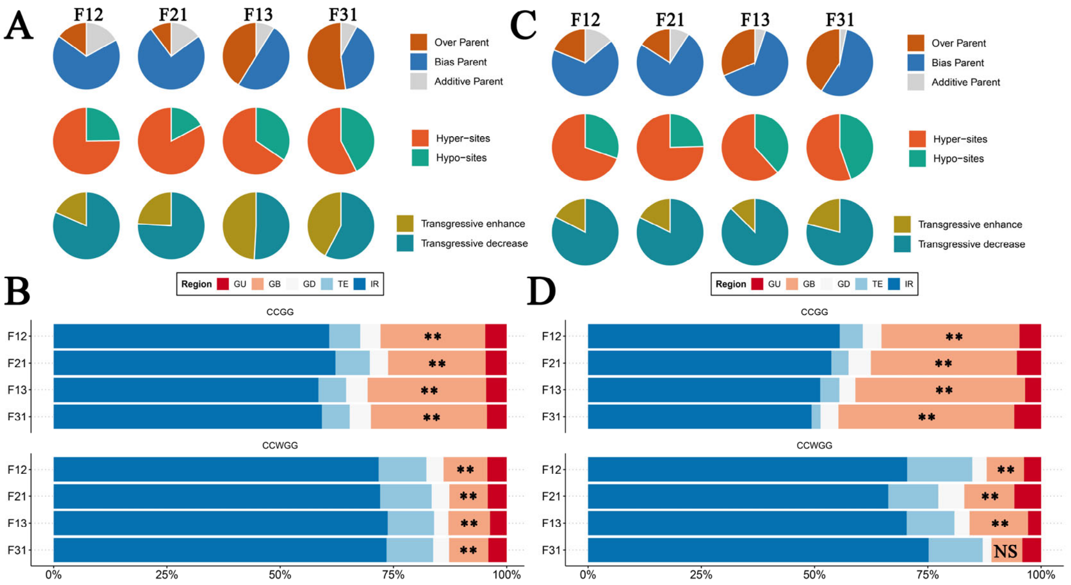

Figure S11 Methylation remodeling patterns in immature seeds of hybrids

(A, C) Classification of DMSs loci within F1 hybrids. A) shows CCGG methylation context and C) shows CCWGG methylation context; (B, D) Distribution of NMSs and AMSs in the genome. GU, upstream 2000 bp of gene body; GD downstream 2000 bp of gene body; GB, gene body; TE, transposon elements; IR, intergenic. In the graphs, "**" indicates a highly significant shift in locus distribution from the natural distribution (Chi-square test, $p < 0.01$) and "NS" indicates no significant shift.

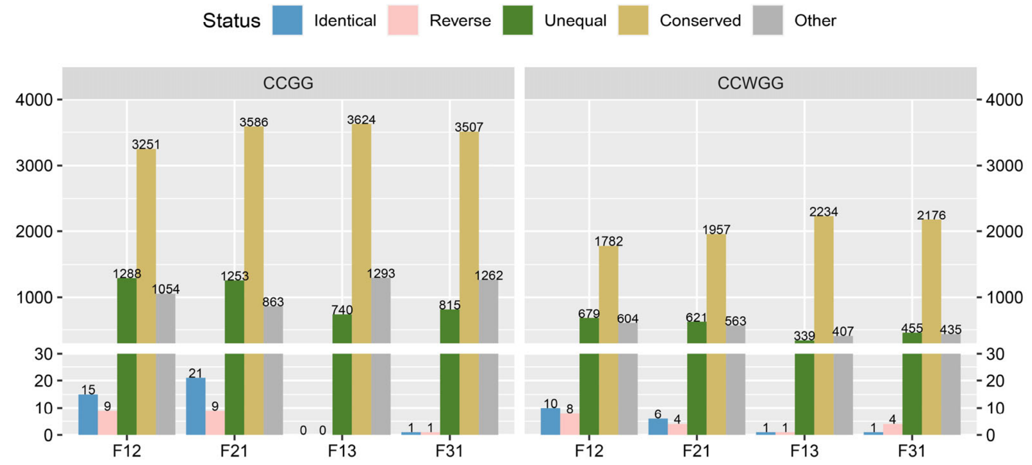

Figure S12 Overlapping genes between methylation and expression change patterns
